# Service readiness and availability of perinatal care in public hospitals - a multi-centric baseline study in Nepal

**DOI:** 10.1186/s12884-022-05121-z

**Published:** 2022-11-15

**Authors:** Dipak Raj Chaulagain, Mats Malqvist, Johan Wrammert, Rejina Gurung, Olivia Brunell, Omkar Basnet, Ashish KC

**Affiliations:** 1 Department of Women’s and Children’s Health, Uppsala Global Health Research on Implementation and Sustainability (UGHRIS), Uppsala University, Dag Hammarskjölds väg 14B, 75185 Uppsala, Sweden; 2 Golden Community, Lalitpur, Nepal; 3 Society of Public Health Physicians Nepal (SOPHPHYN), Kathmandu, Nepal; 4 Department of Women’s and Children’s Health, Uppsala Global Health Research on Implementation and Sustainability (UGHRIS), Uppsala University, Dag Hammarskjölds väg 14B, 751 85 Uppsala, Sweden

**Keywords:** Readiness and availability, Quality improvement, Neonatal care, Scale-up, Nepal

## Abstract

**Background:**

Poor quality of maternal and newborn care contributes to nearly two million deaths of mothers and their newborns worldwide annually. Assessment of readiness and availability of perinatal care services in health facilities provides evidence to underlying bottlenecks for improving quality of care. This study aimed to evaluate the readiness and availability of perinatal care services in public hospitals of Nepal using WHO’s health system framework.

**Methods:**

This was a mixed methods study conducted in 12 public hospitals in Nepal. A cross-sectional study design was used to assess the readiness and availability of perinatal care services. Three different data collection tools were developed. The tools were pretested in a tertiary maternity hospital and the discrepancies in the tools were corrected before administering in the study hospitals. The data were collected between July 2017 to July 2018.

**Results:**

Only five out of 12 hospitals had the availability of all the basic newborn care services under assessment. Kangaroo mother care (KMC) service was lacking in most of the hospitals (7 out of 12). Only two hospitals had all health workers involved in perinatal care services trained in neonatal resuscitation. All of the hospitals were found not to have all the required equipment for newborn care services. Overall, only 60% of the health workers had received neonatal resuscitation training. A small proportion (3.2%) of the newborn infants with APGAR < 7 at one minute received bag and mask ventilation. Only 8.2% of the mothers initiated breastfeeding to newborn infants before transfer to the post-natal ward, 73.4% of the mothers received counseling on breastfeeding, and 40.8% of the mothers kept their newborns in skin-to-skin contact immediately after birth.

**Conclusion:**

The assessment reflected the gaps in the availability of neonatal care services, neonatal resuscitation training, availability of equipment, infrastructure, information system, and governance. Rapid scale-up of neonatal resuscitation training and increased availability of equipment is needed for improving the quality of neonatal care services.

## Background

In 2016, 5.0 million deaths were associated with poor quality of care in low-and-middle-income countries, with maternal and newborn causes constituting a large proportion [1]. Worldwide, poor quality of essential maternal and newborn care contributes to nearly two million deaths of women and their newborns annually [2, 3], and improving the quality of services around the time of birth is critical to reducing mortality and morbidity among women and newborn infants [4, 5]. The Sustainable Development Goals (SDG) urges nations, especially resource-poor countries, to accelerate the implementation of the evidence-based interventions for women and newborns by ensuring quality [6]. The implementation of these interventions in the real-life setting is however not straightforward [7]. Common constraints to scale-up of effective interventions are found in all high-burden countries, with the most important bottlenecks relating to the health workforce, finance, and service delivery [8, 9].

Appropriate measurement of the quality of services is the first step to ensuring the best quality of care for women and newborns [10]. However, the regular assessment of health service delivery has often remained a weak component of national and global monitoring of progress and performance [11]. Assessment of readiness and availability of perinatal care services in health facilities provides evidence to the underlying bottlenecks for improving quality of care [12]. Also, assessing the service readiness of health facilities will help broaden our understanding of their ability to adjust to strategic changes [13]. A number of models have been developed for the measurement of quality of care [4]. The Donabedian model proposes a triad of structure, process, and outcome to evaluate the quality of health care [14]. Safety, effectiveness, timeliness, efficiency, equity, and people-centeredness are the major characteristics of quality of care as defined by the World Health Organization (WHO) and the Institute of Medicine [15]. WHO’s health system framework of quality of care focuses on six building blocks; (i) service delivery, (ii) health workforce, (iii) health information systems, (iv) essential medicines, (v) financing, and (vi) leadership/ governance [4, 16]. Based on the analysis of previously developed models, the WHO has developed a framework to assess and improve maternal and newborn care in health facilities with 8 standards of care, 31 quality statements, and 352 quality measures [4, 10].

Despite the impressive gains in the number of physical facilities and health workforce, delivering quality services to the population remains a challenge in Nepal [17]. A multi-centric study conducted in over 8000 health facilities from ten countries, including Nepal, indicated substantial gaps in the basic capacity to deliver health services [18]. Health facilities across the nation are struggling with the persistent absence of health workers, stock out of drugs and commodities, poorly maintained infrastructures and equipment, insufficient opening hours, and insufficient control of hazardous waste and basic infection prevention practices [17]. In Nepal, the major gaps in maternal, neonatal and Child Health (MNCH) services have been identified in health financing, workforce, essential medical products and technologies, and governance [17]. A low level of competency among health workers has been identified in the management of pre-term newborns, newborn resuscitation, and inpatient care for sick and small children [17]. Several health facilities lack essential equipment to perform newborn resuscitation services; facility readiness to provide quality maternal and newborn care was found to be low in a rural Southern district of Nepal [5]. Nepal Health Facility Survey (NHFS) revealed that only four out of 10 health facilities had carried out neonatal resuscitation and only 3% of Primary Health Care Centers (PHCCs) had performed all basic emergency obstetric signal functions at least once in the three months preceding the survey [19]. To improve the quality of perinatal care, a recent study indicated the need of implementing tailored strategies, including recruitment of health worker, supervision and onsite coaching and access to necessary equipment and medicine in health facilities [20].

Improving the quality of care is one of the four strategic directions adopted by the Nepal Health Sector Strategy 2015–2020 to improve the health status of the population [21]. The results of previous studies in a tertiary maternity hospital in Nepal prompted efforts to scale up and test the adaptability of a quality improvement (QI) package for perinatal care services in other health facilities in Nepal [22, 23]. Based on these results, the Ministry of Health and Population developed a QI intervention model, Nepal Perinatal Quality Improvement Package (NePeriQIP), and scaled it up in 12 public hospitals [24]. To understand the context of perinatal services before initiating the QI intervention, a baseline assessment for perinatal care was conducted in the hospitals. Following this assessment, a bottleneck/causal analysis workshop was conducted in each hospital. Based on this process, this study aims to evaluate the readiness and availability of perinatal care services in public hospitals of Nepal using WHO’s health system framework.

## Methods

### Study Design

This study was part of a large-scale stepped wedge cluster randomized trial (NePeriQIP, ISRCTN: 30,829,654) to improve the quality of perinatal care [25]. The study was conducted from July 2017 to July 2018. A cross-sectional design was used to assess the readiness and availability of perinatal care services in public hospitals in Nepal. A mixed methods design was applied to assess the readiness and availability of perinatal care; the qualitative component was adopted to triangulate the quantitative findings. The data were collected at different time points from different hospitals as per the stepped wedge study design of NePeriQIP [25].

## Study framework

The study utilized a mixed methods framework using both quantitative and qualitative data. For quantitative data, the data collection tools were developed based on the six building blocks of WHO’s health system framework of quality of care; (i) service delivery, (ii) health workforce, (iii) health information systems, (iv) essential medicines, (v) financing and (vi) leadership/governance [4, 16]. A format was developed for the collection of qualitative data.

## Study site

This study was conducted in 12 public hospitals in Nepal. The hospitals were selected by the Ministry of Health and Population with the criteria of having > 1,000 deliveries per year. The hospitals were scattered throughout the country, mostly in the flatlands. All hospitals were referral hospitals for providing maternal and perinatal care. Altogether, the hospitals contributed more than 60,000 deliveries per year at the time of selection [25].

## Data Collection

### Development of data collection tools

The research team developed three different data collection tools; (1) an assessment checklist for service readiness and availability of perinatal care; (2) a format for causal/ bottleneck analysis and onsite planning for perinatal care; and (3) a questionnaire to collect data from the medical records and registries. The data collection tools were developed based on the indicators for assessing service readiness and availability based on the WHO’s health system building blocks, and quality of care framework for maternal and newborn health [4]. To ensure validity and reliability, the data collection tools were developed in consultation with newborn health program managers at the ministry of health and population. Further, the data collection tools were pretested in a tertiary maternity hospital in Kathmandu and the discrepancies in the tools were corrected before administering in the study hospitals.

### Data collection procedure

#### Data collection using the checklist for the assessment of service readiness and availability

To facilitate the implementation of the QI process, each hospital recruited in-hospital QI facilitators from among the pediatricians, medical officers, and nursing staff. In-hospital QI facilitators were mobilized for the collection of data using this checklist in their respective hospitals. The QI facilitators received a one-day training on using the checklist before initiating the data collection process.

#### Data collection using the format for causal/ bottleneck analysis for perinatal care

Following the administration of the checklist for the assessment of service readiness and availability, a two-day bottleneck analysis workshop was organized in the respective hospitals. In-hospital QI facilitators together with the representatives of the study team facilitated the workshop. The health workers from the delivery unit, sick newborn care unit, and emergency and pediatric departments including key managerial staff participated in the workshop. At the start, the in-hospital QI facilitators shared the major findings of the assessment of service readiness and availability in the hospital. The participants of the workshop worked in groups to identify the major problems related to perinatal care in the hospital. Three groups were formed and each group was assigned to discuss problems related to different themes in terms of health system building blocks. The first group discussed and identified problems related to immediate newborn care and neonatal resuscitation. The second group worked on kangaroo mother care (KMC) and breastfeeding, and the third group on infection prevention. The participants used the predefined format to identify and list the identified problems. According to the format, the problems identified by each group were categorized in terms of health system building blocks as follows; (i) availability of services, (ii) human resources, (iii) infrastructure and equipment, (iv) health information system, and (v) governance and financing. The major problems identified by the respective groups were presented using flipcharts in the plenary for discussion. The final list of the problems for each theme was finalized after the discussion. The groups then did root cause analysis for the identified problems. The ‘five whys’ method was used to explore the possible root causes for each problem [26]. The root causes identified by each group were then presented in the plenary using flipcharts. The research team collected the flipcharts for compilation and further analysis.

#### Questionnaire to collect data from medical records and registries

Data on the performance of health workers on neonatal care at baseline was collected from the hospital registry by a team of independent data collectors. The data collection team consisted of nurses experienced in nursing care and data collection. The data collector received a seven-day training before initiating the data collection process. The data were collected in paper formats from all hospitals.

### Data Management and analysis

#### Data collected using the checklist for the assessment of service readiness and availability

After the QI facilitator filled out the checklist for the assessment of readiness and availability, an external mentor verified the checklist for completeness and accuracy. In case of incompleteness and low data quality, QI facilitators reassessed and refilled the checklist. After completing the assessment, the hospital authority reviewed and attested the completed checklist. The data collected through this checklist were then entered by a data entry officer into an electronic database developed in Census and Survey Processing System (CSPro) software.

Descriptive data analysis was applied to the study. Status of service availability, referral services for newborn care, training of health workers on neonatal resuscitation, availability of essential equipment, infrastructure, information system, and governance were analysed for the following services:

- **Immediate newborn care and neonatal resuscitation;** Immediate newborn care interventions include delayed cord clamping and cord cleansing with antiseptics, drying, head covering, skin-to-skin care, delayed bathing, and early initiation and exclusive breastfeeding [27]. Resuscitation care support is required for those newborn infants who do not cry or establish breathing immediately after birth [28].
- **KMC and breastfeeding;** Kangaroo mother care (KMC) is an effective intervention that comprises a set of care practices to save lives of premature and low birth weight newborn infants; continuous skin-to-skin contact, establishing breastfeeding, and close follow up after discharge from health facility [29, 30].

**Fig. 1:**
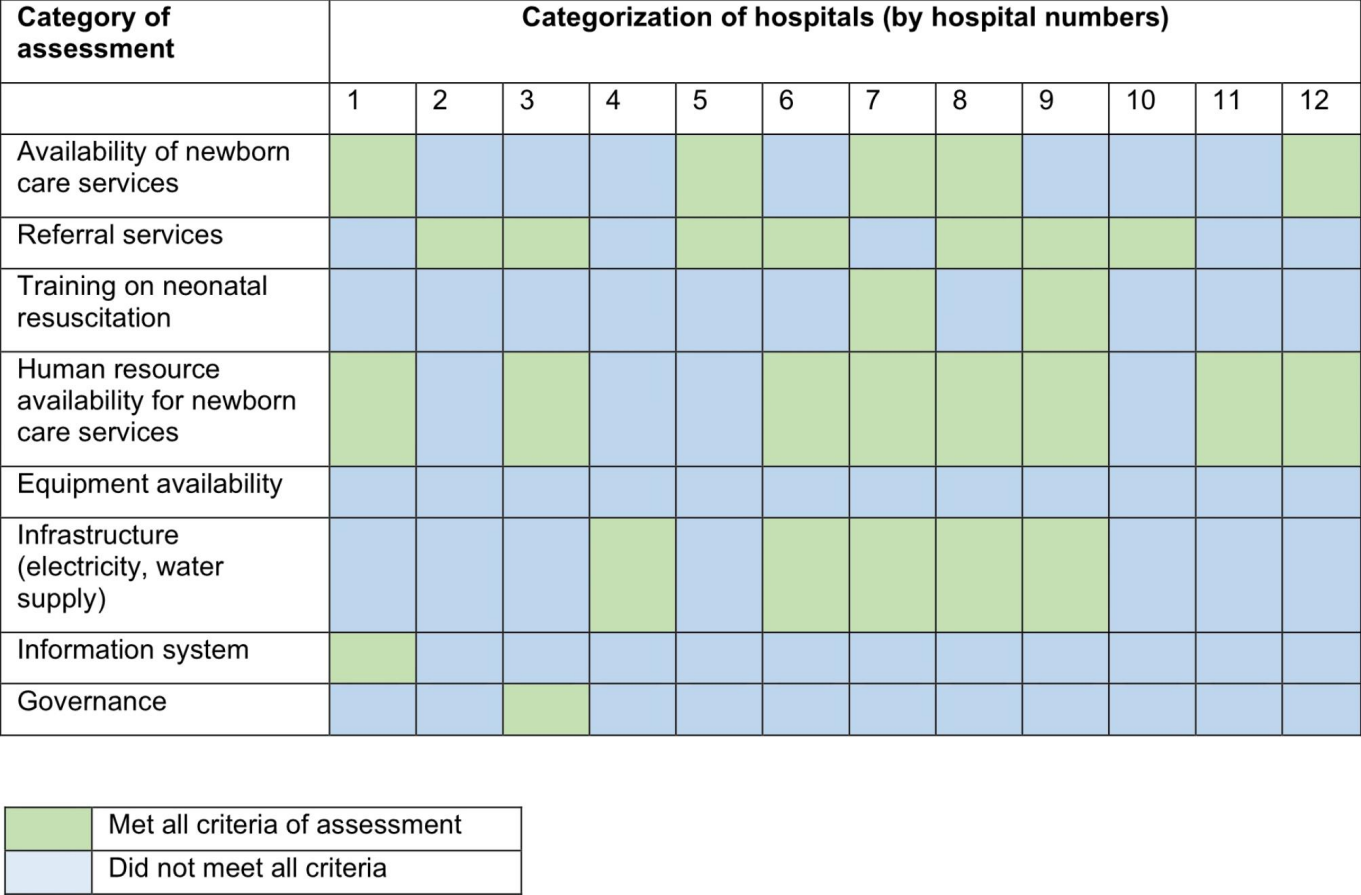
Categorization of hospitals based on the fulfilment of criteria under assessment

The findings were categorized for individual hospitals. The hospitals were assigned different color codes based on the fulfillment of the criteria under each assessment category (Table 1). The hospitals meeting all the criteria under assessment were assigned a light green color, and those which did not meet all the criteria were assigned a light blue color. The findings generated from this analysis have been presented in Fig. 1 and Table 2.

**Table 1:** Category of health system building blocks assessed, and assessment criteria

Category	Assessment criteria
Availability of services	• Twenty-four hours delivery services, immediate newborn care services, KMC, breastfeeding support, and sick newborn care services
Referral services	• Functional ambulance or other vehicles on-site for referrals, availability of fuel in the vehicle, mechanism or system to make phone calls available all the time, and availability of mobile phone/landline numbers on-site for communication during the referral
Training on neonatal resuscitation	• Number of health workers who received training on neonatal resuscitation before
Availability of human resource for newborn care services	• Availability of duty roster (24-hour clock), availability of staffs on their designated shifts, availability of a skilled person in conducting deliveries present at the hospital or on-call round the clock, including weekends and public holidays to provide delivery service, availability of obstetrician to respond to complicated deliveries 24 h a day, and availability of pediatrician to respond to complicated deliveries 24 h a day
Availability of essential equipment	• **Monitoring equipment** (stethoscope with neonatal chest piece, non-invasive BP monitors, heart rate/apnea monitor, pulse oximeter, low reading clinical thermometer, room thermometer, electronic baby weighing scale, mechanical baby weighing scale) • **Equipment for management** (radiant warmer, phototherapy unit, CPAP, ventilator, glucometer) • **Resuscitation equipment** (self-inflating bags, foot-operated suction pumps/ mucus traps, penguin suction, bag and mask)
Infrastructure (electricity, water supply)	• **Designated area for;** labor room, operating theatre, postnatal ward, newborn corner, KMC corner/unit, SNCU/NICU, breastfeeding corner, hand-washing space (labor ward), hand-washing space (SNCU/NICU), boiling and autoclaving, laundry, clean utility room, soiled utility room, store, laboratory room, doctor’s room, nurses room • Any in-patient in the maternity ward shared beds at any time before or after delivery in the last three months • Any patients in the maternity ward slept on the floor in the last three months • Any maternity patient delivered on the floor in a corridor or bathroom due to lack of beds in the last three months
Information system	• Routine audits or case reviews on maternal deaths • Routine audits or case reviews on newborn deaths/stillbirths • System in place to regularly collect maternal and neonatal health service data • Display of newborn care related data
Governance	• Availability of hospital governance committee • Frequency of the meetings of hospital governance committee in a year • Number of work/action plans created by hospital governance committee

**Table 2:** Training on neonatal resuscitation and availability of equipment

Hospitals	Annual delivery, 2016	Training on neonatal resuscitation			Availability of equipment for newborn care services		
		**Total health workers eligible for neonatal resuscitation training**	**Total health workers who received training on neonatal resuscitation**	**% received training**	**Total items assessed**	**Total items available**	**% of equipment available**
Hospital 1	9427	200	85	42.5	21	18	85.7
Hospital 2	1033	8	7	87.5	21	10	47.6
Hospital 3	11,318	96	51	53.1	21	18	85.7
Hospital 4	1438	17	4	23.5	21	11	52.3
Hospital 5	1065	17	8	47	21	10	47.6
Hospital 6	3139	25	20	80	21	18	85.7
Hospital 7	4276	27	27	100	21	16	76.1
Hospital 8	5767	10	2	20	21	16	76.1
Hospital 9	9007	62	62	100	21	17	80.9
Hospital 10	1194	20	11	55	21	16	76.1
Hospital 11	3280	43	35	81.3	21	14	66.6
Hospital 12	8355	55	38	69	21	17	80.9
Total	59,299	580	350	60.3	252	181	71.8

#### Data collected using the format for causal/ bottleneck analysis for perinatal care

The findings of the bottleneck analysis workshop, collected in flipcharts, were entered into Microsoft Excel for analysis. The data collected in flipcharts from the bottleneck analysis workshop were analysed and the most common problems and root causes were identified. The findings were summarised in a table in terms of major problems and root causes (Tables 3 and 4). The tables represent the collective views of health workers that were documented and presented during the bottleneck analysis workshop after consensus in respective groups.

**Table 3:** The baseline performance of hospitals on bag and mask ventilation among newborns with APGAR < 7 at one minute

Hospitals	Bag and mask ventilation among newborns with APGAR < 7 at one minute (%)
Hospital 1 (n = 1388)	1.8
Hospital 2 (n = 345)	4.9
Hospital 3 (n = 37)	0
Hospital 4 (n = 1002)	3.3
Hospital 5 (n = 347)	0
Hospital 6 (n = 83)	1.2
Hospital 7 (n = 1255)	6.5
Hospital 8 (n = 479)	0.6
Hospital 9 (n = 53)	5.7
Hospital 10 (n = 1072)	8.8
Hospital 11 (n = 2642)	0.6
Hospital 12 (n = 210)	5.2
Total (n = 8913 )	3.2

**Table 4:** Major problems and root causes for immediate newborn care and neonatal resuscitation (based on the presentation from health workers during bottleneck analysis workshop)

Major Problems	Root Cause
1. All health workers not being able to perform appropriate neonatal resuscitation	1. Many health workers not trained in neonatal resuscitation 2. Lack of positive attitude 3. Lack of initiation from health workers 4. Overburden of duty due to inadequate number 5. Low level of interest/ motivation
2. Inadequate equipment for neonatal resuscitation service	1. Unavailability of equipment needed for resuscitation 2. Equipment not functioning (radiant warmer, pulse oximeter) 3. Equipment not available (CPAP, neonatal ventilator)
3. Inadequate hand-washing practice by health workers	1. Sink not available 2. Inadequate space 3. Old/ improper infrastructure 4. Negligence of service providers
4. Low quality of neonatal resuscitation services	1. Adequate space to practice neonatal resuscitation unavailable 2. No supervision and monitoring of resuscitation practice 3. Improper sterilization of Ambu- bag
5. Poor referral mechanisms	1. Lack of training for health workers 2. Inadequate communication with higher-level health facilities
6. Lack of routine reviews	1. Maternal and Perinatal Death Review Committee not functioning 2. Lack of hospital governance committee
7. Poor recording and reporting system	1. Inadequate coordination problem between record department and others 2. Resources are not enough for a health information system 3. Training is not given to all staff according to need for recording reporting

#### Data collected using a questionnaire to collect data from medical records and registries

Data collected through registers and medical records were transferred into CSPro by a team of independent data entry officers. The forms were indexed for respective hospitals before being entered into the database. Descriptive statistics were used to analyse the baseline performance of health workers in neonatal care. This paper focused on some of the representative newborn care services to assess the baseline performances of individual hospitals (bag and mask ventilation (BMV) among newborn infants with APGAR < 7, initiation of breastfeeding among newborns before transfer to post-natal ward, counseling of mothers on breastfeeding and skin-to-skin contact immediately after birth). The findings of the baseline assessment have been presented in Table 2.

The Statistical Package for the Social Sciences (SPSS) version 25.0 was used for all analyses.

## Results

The completed checklists on readiness and availability of newborn care services and the flipcharts presented during the bottleneck analysis workshop were collected from all of the participating hospitals. A total of 8,913 newborn infants with APGAR < 7 at one minute were enrolled for baseline assessment of BMV and 23,143 for assessment of performance on initiation of breastfeeding, skin-to-skin contact, and drying and stimulation.

The availability of basic newborn care services, training on neonatal resuscitation, referral services, availability of equipment for newborn care services, information system, and the governance system were the areas with problems in most of the hospitals (Fig. 1). Only five out of 12 hospitals had the availability of all the basic newborn care services under assessment. Among those hospitals that did not fulfill all the criteria, KMC service was lacking in most of them (7 out of 12 hospitals). One hospital (hospital 10) did not have a sick newborn care service. Only two of the hospitals had all health workers working in perinatal care services trained on neonatal resuscitation. All of the hospitals were found not to have all the required equipment for newborn care services. Only one hospital fulfilled all the criteria of assessment for the information system and so was the situation for the governance system (Fig. 1).

Regarding training on neonatal resuscitation, only 60% of the health workers involved in perinatal care services had received training on neonatal resuscitation (Table 2). The proportion of health workers receiving neonatal resuscitation training ranged from 20% (hospital 8) to 100% (hospital 9). Overall, only 71.8% of the newborn care equipment was available in the hospitals. The percent availability of the required equipment was as low as 47.6% (in two hospitals) to as high as 85.7% (in three hospitals) (Table 2).

The baseline performance of hospitals on the bag and mask ventilation (BMV) has been depicted in Table 4. Overall, only 3.2% of the newborns with APGAR < 7 at one minute received BMV at the time of assessment. The proportion of BMV among newborns with APGAR < 7 at one minute was found to be as low as 0% in two hospitals and the highest proportion was found in hospital 10 (8.8%). Overall, only 8.2% of the mothers initiated breastfeeding to newborn infants before transfer to the post-natal ward and 73.4% of the mothers received counseling on breastfeeding (Table 5). Only 40.8% of the mothers kept their newborn infants in skin-to-skin contact immediately after birth (Table 5).

**Table 5:** Major problems and root causes for kangaroo mother care and breastfeeding (based on the presentation from health workers during bottleneck analysis workshop

Major Problems	Root Cause
1. Kangaroo Mother Care service not initiated in the hospital	1. No separate space to initiate KMC; KMC corner 2. Unavailability of materials, logistics to initiate KMC (KMC cloth) 3. Inadequate human resource 4. Lack of initiation by service providers 5. Low level of motivation
2. Inadequate knowledge about the importance and technique of KMC	1. Untrained service provider
3. Lack of separate space for breastfeeding to ensure privacy	1. No adequate space due to old infrastructure.
4. Early initiation of breastfeeding not sufficient	1. No counseling to mothers by health workers due to workload 2. Health workers do not realize the importance of early initiation of breastfeeding. 3. Practice bottle feeding/ formula feeding 4. Mothers not able to breastfeed within 1 h

The major problems and root causes for immediate newborn care and neonatal resuscitation are shown in Table 3. The major root causes identified by the participants during the bottleneck analysis workshop were; lack of training, lack of positive attitude, lower level of motivation, overburden of duty due to the inadequate number of staff, unavailability of required equipment, poor infrastructure, weak supervision and monitoring, and poor governance mechanism. Similarly, the major root causes related to poor performance on KMC and breastfeeding were related to; infrastructure, required logistics, inadequate number of staff, low level of motivation of health workers, and lack of training (Table 6).

**Table 6:** Baseline performance of hospitals on initiation of breastfeeding, skin-to-skin contact, and drying and stimulation

Hospitals	Initiation of breastfeeding among newborns before transfer to post-natal ward (%)	Mothers counselled on breastfeeding (%)	Skin-to-skin contact immediately after birth (%)
Hospital 1 (n = 355)	11.5	100	82.5
Hospital 2 (n = 397)	14.6	45	63
Hospital 3 (n = 127)	1.5	87.4	95.3
Hospital 4 (n = 3353)	6.1	85.4	7.2
Hospital 5 (n = 2404)	7.2	73.8	60.2
Hospital 6 (n = 414)	3.1	81.4	3.9
Hospital 7 (n = 3573)	17	52.1	18.8
Hospital 8 (n = 1570)	11.9	63.2	7.8
Hospital 9 (n = 423)	11.6	79.4	46.8
Hospital 10 (n = 5612)	9.7	32.9	74.4
Hospital 11 (n = 3807)	2.5	97.2	17
Hospital 12 (n = 1108)	1.9	83.2	13.2
Total (23,143)	8.2	73.4	40.8

## Discussion

To our knowledge, this is the first large-scale mixed method study in Nepal to assess the readiness and availability of perinatal care services in hospitals before the scale-up of a QI intervention model. We found that the KMC service was not available in most of the hospitals. The quantitative analysis showed that around 40% of the health workers involved in perinatal care had not received training on neonatal resuscitation, and inadequacy of the equipment for basic newborn care services was observed in all hospitals. Bag and mask ventilation was performed only on 3.2% of the newborn infants with an APGAR < 7 at one minute.

Regular assessment of hospitals on readiness and availability of health services provides a stimulus for better health services being offered [4, 31]. The color code categorization of hospitals showed gaps in service availability, training of human resources, availability of equipment, infrastructure, information system, and governance. The finding is similar to previous studies related to the readiness and availability of perinatal care services in different settings. An analysis of 12 countries that account for the majority of global maternal and newborn deaths reflected that the health system building blocks with major bottlenecks were health financing, health workforce, and health service delivery [3].

Kangaroo mother care is a simple and cost-effective intervention to prevent mortality and morbidity among premature and low birth weight newborns [30]. We found that the KMC service was not initiated in 58.3% of hospitals (7 out of 12) under assessment in our study. The proportion is lower than the finding of a study in Malawi where 79% of the hospitals reported having inpatient KMC services [29]. WHO recommends KMC for stable newborns ≤ 2000 g as an evidence-based intervention to improve preterm birth outcomes [4, 29]. Rapid scale-up of this cost-effective intervention is indicated in hospitals in Nepal and other hospitals in similar settings [17].

Regular training on neonatal resuscitation is key to upgrade and retain knowledge and skills on neonatal resuscitation [32, 33]. A substantial proportion of health workers lacking training on neonatal resuscitation in our study resembles the findings by Kathryn et al. in Zambia [11]. The availability of staff who had been trained in the integrated management of pregnancy and childbirth in the preceding two years was generally low in Zambia [11]. The finding suggested the need to scale up neonatal resuscitation training in hospitals for the improved outcome of neonatal resuscitation care services.

Delivery of quality newborn care services is not possible without having required equipment in place [4]. None of the hospitals under assessment in our study had the complete set of required equipment for neonatal care. Out of the total 21 equipment assessed in each hospital, the maximum number of items available was 18 which was observed in three hospitals. Two of the hospitals had only 10 equipment available at the time of assessment. This gap in the availability of equipment indicates a lower level of readiness to cater neonatal services. The finding is similar to the study conducted in Malawi where notable gaps were observed for items related to infection prevention and thermal control, which are directly linked to sepsis and preterm birth [34]. Similarly, a study in Zambia showed that on average, health facilities had nine of the 14 tracer items indicating an overall readiness score of 61% [11]. The mean availability of the tracer items for Basic Emergency Obstetric and Neonatal Care (BEmONC) service was found to be 60.4% at referral hospitals in Madagascar [35].

The gaps observed in the training of health workers and availability of equipment were reflected in the lower level of performance on selected newborn care services in the hospital. The quantitative findings were triangulated by the collective views of health workers related to perinatal care in participating hospitals. The most common problems and root causes identified by the health workers were related to training, equipment, and infrastructure. The other problem and root cause was lack of initiation from health workers.

This study has two major strengths. First, this study was conducted in the 12 different hospitals representing the existing level of government hospitals in Nepal. Therefore, the findings are fairly generalizable in other hospitals in Nepal and other similar settings. Second, we have used both quantitative and qualitative data to validate the findings that provide a deeper understanding of the existing gaps in service readiness and availability in the hospitals.

There are three major limitations of the study. First, the quantitative analyses were limited to descriptive statistics only. Second, we did not cover all newborn care components for analysis of baseline performance. Only selected newborn care services were analysed to represent the overall performance of newborn care services. Third, we did not cover all components of the health system building block for analysis, particularly the health financing component.

## Conclusion

The assessment of these 12 hospitals in Nepal reflected gaps in neonatal care service availability, training of health workers, availability of equipment, infrastructure, information system, and governance. Rapid scale-up of neonatal resuscitation training and increased availability of equipment is needed to improve the quality of neonatal care services in public hospitals. Further studies can focus on a detailed analysis of information systems and governance to improve neonatal care services. Regular assessment of hospitals and health facilities is indicated to understand the existing gaps in health system building blocks, and for continuous improvement of quality of care.

## Data Availability

The datasets used and analysed for the study are available from the corresponding author on reasonable request.

## Abbreviations

APGAR: Appearance, Pulse, Grimace, Activity, and Respiration
BMV: Bag and mask ventilation
CSPro: Census and Survey Processing System
ISRCTN: International Standard Randomised Controlled Trial Number
KMC: Kangaroo Mother Care
NePeriQIP: Nepal Perinatal Quality Improvement Project
QI: Quality Improvement
SDG: Sustainable Development Goals
WHO: World Health Organization
UGHRIS: Uppsala Global Health Research on Implementation and Sustainability
